# Targeted therapy of advanced parathyroid carcinoma guided by genomic and transcriptomic profiling

**DOI:** 10.1002/1878-0261.13398

**Published:** 2023-04-11

**Authors:** Maria‐Veronica Teleanu, Carmina T. Fuss, Nagarajan Paramasivam, Sebastian Pirmann, Andreas Mock, Christoph Terkamp, Stefan Kircher, Laura‐Sophie Landwehr, Christina Lenschow, Nicolas Schlegel, Albrecht Stenzinger, Arne Jahn, Martin Fassnacht, Hanno Glimm, Daniel Hübschmann, Stefan Fröhling, Matthias Kroiss

**Affiliations:** ^1^ Division of Translational Medical Oncology, National Center for Tumor Diseases (NCT) Heidelberg German Cancer Research Center (DKFZ) Heidelberg Germany; ^2^ Department of Internal Medicine I, Division of Endocrinology and Diabetes, University Hospital University of Würzburg Germany; ^3^ Computational Oncology Group, Molecular Precision Oncology Program NCT Heidelberg and DKFZ Germany; ^4^ Department of Gastroenterology, Hepatology and Endocrinology Hannover Medical School Germany; ^5^ Institute of Pathology University of Würzburg Germany; ^6^ Department of General, Visceral, Transplant, Vascular and Pediatric Surgery, University Hospital University of Würzburg Germany; ^7^ Institute of Pathology Heidelberg University Hospital Germany; ^8^ Institute for Clinical Genetics, Faculty of Medicine Carl Gustav Carus Technische Universität Dresden Germany; ^9^ ERN‐GENTURIS, Hereditary Cancer Syndrome Center Dresden Germany; ^10^ Comprehensive Cancer Center Mainfranken University of Würzburg Germany; ^11^ Department of Translational Medical Oncology National Center for Tumor Diseases (NCT/UCC) Dresden Germany; ^12^ German Cancer Research Center (DKFZ) Heidelberg Germany; ^13^ Faculty of Medicine and University Hospital Carl Gustav Carus Technische Universität Dresden Germany; ^14^ Helmholtz‐Zentrum Dresden – Rossendorf (HZDR) Germany; ^15^ Translational Medical Oncology, Faculty of Medicine and University Hospital Carl Gustav Carus Technische Universität Dresden Germany; ^16^ Translational Functional Cancer Genomics National Center for Tumor Diseases (NCT) and German Cancer Research Center (DKFZ) Heidelberg Germany; ^17^ German Cancer Consortium (DKTK) Dresden Germany; ^18^ DKTK Heidelberg Germany; ^19^ Department of Internal Medicine IV, University Hospital Ludwig Maximilians University Munich Germany

**Keywords:** immune checkpoint inhibition, mutational signature, RNA sequencing, tumour mutational burden, tyrosine kinase inhibition, whole‐genome sequencing

## Abstract

Parathyroid carcinoma (PC) is an ultra‐rare malignancy with a high risk of recurrence after surgery. Tumour‐directed systemic treatments for PC are not established. We used whole‐genome and RNA sequencing in four patients with advanced PC to identify molecular alterations that could guide clinical management. In two cases, the genomic and transcriptomic profiles provided targets for experimental therapies that resulted in biochemical response and prolonged disease stabilization: (a) immune checkpoint inhibition with pembrolizumab based on high tumour mutational burden and a single‐base substitution signature associated with APOBEC (apolipoprotein B mRNA editing enzyme, catalytic polypeptide‐like) overactivation; (b) multi‐receptor tyrosine kinase inhibition with lenvatinib due to overexpression of *FGFR1* (Fibroblast Growth Factor Receptor 1) and *RET* (Ret Proto‐Oncogene) and, (c) later in the course of the disease, PARP (Poly(ADP‐Ribose) Polymerase) inhibition with olaparib prompted by signs of defective homologous recombination DNA repair. In addition, our data provided new insights into the molecular landscape of PC with respect to the genome‐wide footprints of specific mutational processes and pathogenic germline alterations. These data underscore the potential of comprehensive molecular analyses to improve care for patients with ultra‐rare cancers based on insight into disease biology.

## Graphical abstract

Our case study on advanced parathyroid carcinoma using whole genome and transcriptome sequencing improved our understanding of the molecular pathogenesis of the disease by identifying a relevant contribution of the APOBEC signatures SBS2 and SBS13 to the mutational process, as well as a *MUTHY* heterozygous germline mutation as a potential predisposing factor. Therapeutically, transcriptome sequencing enabled the detection of recurrent *FGFR1* and *RET* overexpression and guided targeted therapy.

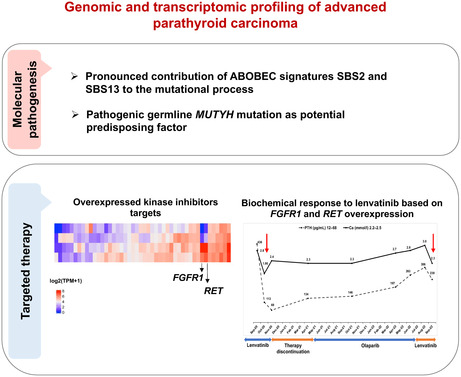

**Abbreviations**
APOBEC: apolipoprotein B mRNA editing enzyme catalytic polypeptide‐like
CC: cell cycle
CNV: copy number variation
DDR: DNA damage repair
DEV: developmental regulation
DKFZ: German Cancer Research Center
DKTK: German Cancer Consortium
FGFR1: fibroblast growth factor receptor 1
IE: immune evasion
indel: small insertions and deletion
LN: lymph node
LOH‐HRD: loss of heterozygosity‐homologous recombination deficiency
LST: large‐scale state transition
MASTER: Molecularly Aided Stratification for Tumor Eradication Research
MSI: microsatelite instability
MTB: molecular tumour board
NCT: National Center for Tumor Diseases
OTH: other
PAM: PI3K‐AKT‐mTOR
PARP: poly(ADP‐ribose) polymerase
PC: parathyroid carcinoma
RET: Ret proto‐oncogene
RME: RAF‐MEK‐ERK
RNA‐seq: RNA sequencing
SBS3: single‐base substitution signature 3
SNV: single‐nucleotide variant
SV: structural variation
TK: tyrosine kinase
TMB: tumour mutational burden
TPM: transcript per million
WGS/WES: whole‐genome/exome sequencing

## 1 Introduction

Parathyroid carcinoma (PC) is an ultra‐rare malignancy with an incidence of approximately 0.03 per 100 000 persons per year [1, 2]. In the vast majority, tumoural parathyroid hormone secretion results in hypercalcemia, which can be life‐threatening. PC accounts for < 1% of all cases of primary hyperparathyroidism [3]; however, the diagnosis is rarely made during preoperative workup and is based on histopathology with capsular and vascular invasion as the main features of malignancy. Surgery is the only curative treatment for PC [4].

In a retrospective study of 83 patients with PC, disease‐specific overall survival was favourable, but nearly 40% of patients experienced recurrence. Factors associated with longer recurrence‐free survival were low tumour stage, a Ki67 index < 10%, normalization of calcium levels after surgery and absence of lymph node (LN) invasion [5]. Recurrence is locoregional in most cases, and surgery is the mainstay of treatment but may lead to severe complications such as recurrent laryngeal nerve palsy [6]. Distant metastasis occurs in < 10% of patients [5]. To date, there is no systemic treatment for patients with locally advanced or metastatic PC except for the calcimimetic cinacalcet to control hypercalcemia [7].

Known molecular drivers of PC include germline and somatic mutations in *CDC73* and *MEN1*. Furthermore, activation of the PI3K‐AKT‐mTOR (PAM) pathway and amplification of *CCND1* have been observed in up to one‐third of patients [8, 9, 10, 11, 12, 13, 14]. Despite genome‐wide studies that have led to a better molecular understanding of PC, clinically actionable genomic or transcriptomic alterations have not been reported [7].

The National Center for Tumor Diseases (NCT), the German Cancer Research Center (DKFZ) and the German Cancer Consortium (DKTK) conduct MASTER (Molecularly Aided Stratification for Tumor Eradication Research), a prospective observational study that applies whole‐genome/exome sequencing (WGS/WES) and RNA sequencing (RNA‐seq) to inform the care of young adults with advanced malignancies and patients with incurable rare cancers [15, 16]. Here, we describe the molecular profiles of four patients with advanced PC enrolled in MASTER and demonstrate, for the first time, the utility of broad molecular profiling for the clinical management of this disease, including the application of targeted treatment strategies.

## 2 Materials and methods

### 2.1 Patients

Metastases of four patients aged 39–82 years with advanced PC and imminent need for systemic treatment, followed between 2017 and 2020 by two tertiary referral centres, were studied by WGS and RNA‐seq as part of NCT/DKFZ/DKTK MASTER. In all cases, peripheral blood was used as a control. The trial was approved by the Ethics Committee of Heidelberg University (protocol no. S‐206/2011), and all patients provided written informed consent. Clinical data were collected as part of clinical routine. The study was conducted in accordance with the Declaration of Helsinki.

### 2.2 Sample collection, molecular profiling, bioinformatics analysis, and clinical decision‐making

Sample processing was done as previously published [17]: A fresh‐frozen tumour specimen and matched peripheral blood were collected from each patient. Samples were pseudonymized, and tumour histology and cellularity were assessed before further processing. DNA and RNA from the tumour specimen and DNA from the blood sample were isolated using the AllPrep DNA/RNA/Protein Mini Kit (Qiagen, Hilden, Germany), followed by quality control and quantification using a Qubit 2.0 Fluorometer (Life Technologies, Darmstadt, Germany), a 2200 TapeStation system (Agilent, Waldbronn, Germany) and a 2100 Bioanalyzer system (Agilent). WGS and RNA‐seq libraries were prepared using the TruSeq Nano LT DNA Sample Prep Kit (Illumina, San Diego, CA, USA) and the TruSeq RNA Sample Preparation Kit (Illumina), and paired‐end sequencing was carried out with a HiSeq X or HiSeq 4000 instrument (Illumina).

Mapping and alignment of sequencing reads, detection of somatic single‐nucleotide variants (SNVs), small insertions and deletions (indels), DNA structural variations (SVs) and copy number variations (CNVs), and assessment of gene expression were performed using previously reported bioinformatics workflows [15, 18, 19, 20, 21]. A variant frequency > 1% in the DKFZ local control database consisting of 4879 WGS and 1198 WES samples was used to remove common artefacts and single‐nucleotide polymorphisms from the somatic SNVs and indels. Supervised mutational signature analysis of somatic SNVs was performed using yapsa (version 1.14.0, R version 4.0.0) on cosmic v3 signatures [22]. Genomic instability was assessed using the loss‐of‐heterozygosity‐homologous‐recombination‐deficiency (LOH‐HRD) score and the number of large‐scale state transitions (LSTs) as previously described [23, 24, 25]. Microsatellite instability was assessed using the msisensor algorithm [26]. Clustering of samples based on somatic CNVs was performed using the pairwise Jaccard distance calculated from somatic gains and losses. Germline variants were analysed based on joint calling of tumour and control samples using platypus (version 0.8.1.1) and further annotated with gnomad (version 2.1) and the local controls database using vcfanno (version 0.3.2), and variants with a minor allele frequency > 0.005 in gnomad and a frequency > 0.05 in the local control database were removed. vep (version 97) was used to annotate the rare variants in detail. Rare germline variants were evaluated according to American College of Medical Genetics and Genomics and Association for Molecular Pathology criteria and further specifications [27, 28]. Mappings of control sequencing of *MEN1* and *CDC73* were manually inspected and did not indicate structural variants. Immune infiltration analysis was performed using transcript per million (TPM) values with seven different tools via the unified interface of immunedeconv (version 2.0.2) [29].

The progeny algorithm (R package progeny version 1.12.0) [30] was used to estimate, based on TPM values determined by RNA‐seq, the activity states of 14 signalling pathways involved in tumorigenesis (Androgen, EGFR, Estrogen, Hypoxia, JAK‐STAT, MAPK, NFkB, p53, PI3K, TGFb, TNFa, Trail, VEGF, WNT). Based on RNA‐seq data, small‐molecule inhibitors were selected and prioritized according to the number of overexpressed kinases. The targets of clinical kinase inhibitors were derived from Klaeger et al. [31] using an affinity cut‐off of 1 μm to consider only high‐affinity binding. A kinase gene was considered overexpressed if the expression was higher than that of 75% of all kinase genes across all samples (Tables S1 and S2).

Biological curation and clinical annotation of genomic and transcriptomic alterations were performed as previously described [15], and treatment recommendations were determined within the interdisciplinary molecular tumour board (MTB) at NCT Heidelberg, involving the treating physicians.

## 3 Results

### 3.1 Genomic landscape

Key clinical characteristics are provided in Table 1. All patients had undergone multiple surgical treatments and received cinacalcet for calcium control. WGS identified a median of 92 functional somatic SNVs (PC‐A, 496; PC‐B, 45; PC‐C, 14; PC‐D, 139) and a median of nine functional somatic indels (PC‐A, 7; PC‐B, 18; PC‐C, 1; PC‐D, 11). Across all patients, 14 of 731 functional somatic mutations affected known and candidate PC driver genes [8, 10, 11] (Fig. S1, Table S3).

**Table 1:** Clinical characteristics. Gy, Gray; N/A, not available.

Parameter	PC‐A	PC‐B	PC‐C	PC‐D
Age at diagnosis, years	43	51	39	82
Sex	Female	Male	Male	Female
Follow‐up from diagnosis, months	200	260	312	31
Calcium at diagnosis, mmol·L^−1^ (reference range, 2.0–2.7)	3.6	3.6	N/A	3.5
Parathyroid hormone at diagnosis, pg·mL^−1^ (reference range, 12–65)	6770	321	N/A	883
LN metastasis at diagnosis	Yes	No	No	No
Distant metastasis at diagnosis	No	No	No	No
Primary surgery	Left parathyroidectomy, hemithyroidectomy, central LN dissection	Right parathyroidectomy, hemithyroidectomy, central LN dissection	Left parathyroidectomy, hemithyroidectomy	Right parathyroidectomy, hemithyroidectomy
Resection status	R0	N/A	N/A	RX
Postoperative external beam radiation	Yes (thyroid and LN; cumulative dose, 50.4 Gy)	No	No	No
Postoperative recurrence, years after diagnosis	12	4	13	1
Metastasis at relapse	Abdominal LN^a^ (omentum minus and liver hilus)	LN, lung, skull base^a^	Cervical LN^a^	Cervical LN^a^, lung
Surgery at recurrence	Yes	Yes	Yes	Yes
Medication with calcimimetics	Yes	Yes	Yes	Yes

^a^ Sample analysed in this study.

**Fig. 1:**
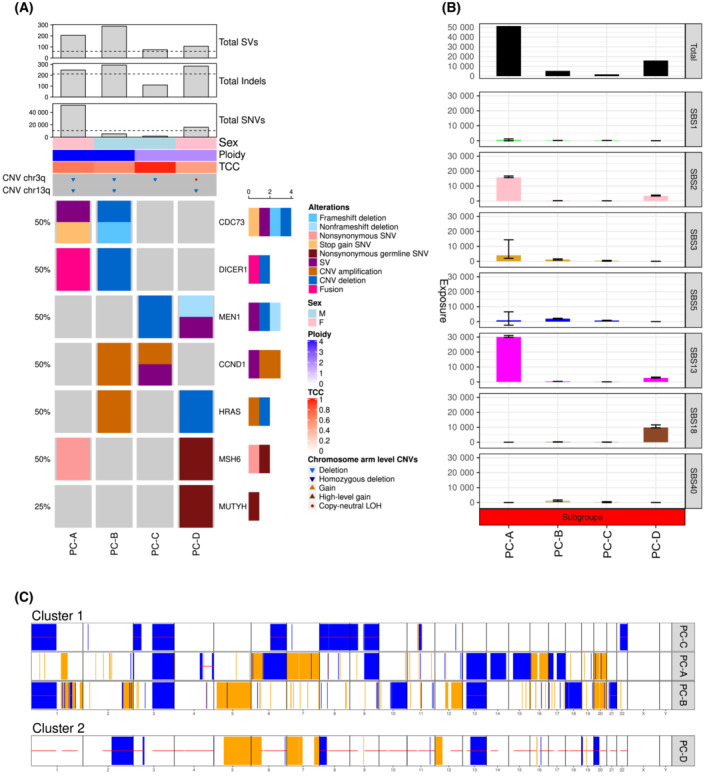
Genomic landscape of PC. (A) Recurrent (identified in at least two samples) mutations affecting known PC driver genes and germline predisposition genes (from a curated list of 143 established predisposition genes). (B) Absolute contribution of mutational signatures to the overall SNV load in PC patients. Each bar represents the number of SNVs explained by the respective mutational signature in an individual tumour. Error bars represent 95% confidence intervals. SBS1, clock‐like, spontaneous deamination; SBS2 and SBS13, altered APOBEC activity; SBS3, defective HR; SBS18, damage by reactive oxygen species. Of note, SBS18 shows a similar profile as SBS36, which is associated with defective base excision repair due to *MUTYH* inactivation. In patient PC‐D, who displays SBS18, a pathogenic *MUTYH* germline mutation was identified. SBS40 is of unknown aetiology, but mutation numbers attributed to this signature are correlated with patient age in some cancer types. (C) Two CNV clusters with loss of chromosome 3q in patients PC‐A, B, and C. TCC, tumour cell content.

Six genes (*CDC73*, *MEN1*, *MSH6*, *CCND1*, *DICER1* and *HRAS*) were altered in more than one case (Fig. 1A). PC‐A harboured two inactivating *CDC73* mutations (p.Y97* and p.W32Dfs*21). PC‐B had biallelic *CDC73* inactivation due to a *CDC73* K34Rfs*3 frameshift deletion and LOH. PC‐C showed a deletion with LOH at chromosome 11q13.1, including *MEN1*. PC‐D harboured a *MEN1* p.Q547_S561delinsH in‐frame deletion. *MSH6* was affected by two somatic missense mutations (p.D135N and p.S574L) in PC‐A, which were classified as variants of unknown significance. Immunohistochemistry showed no loss of mismatch‐repair protein expression in this case, suggesting benign mutations. *CCND1* was amplified in PC‐B and PC‐C. Outlier overexpression of *CCND1* was observed in PC‐C and PC‐A, but we observed no somatic or germline alterations affecting *CCDN1* in PC‐A (Fig. S2). *HRAS* and *DICER* were affected by CNVs or SVs in two samples each.

In one case each, we found a p.E2181K missense mutation in the phosphatidylinositol 3‐ and 4‐kinase domain of *MTOR* that has not been functionally characterized, a p.S183X stop‐gain mutation in the DNA‐binding domain of *TP53*, a functionally unannotated p.H483Y missense mutation in *RB1* and a p.S567X stop‐gain mutation in *ATRX*. In two patients, PC‐A and PC‐D, tumour mutational burden (TMB) was considered high [15], with 503 and 150 functional somatic SNVs and indels.

Single‐base substitution signature 3 (SBS3), which is highly prevalent in HRD‐related cancers [22], was detected in three patients (Fig. 1B). SBS2 and SBS13, associated with overactivation of the physiologically mutagenic enzyme APOBEC (apolipoprotein B mRNA editing enzyme, catalytic polypeptide‐like) [22], were detected in all patients, with PC‐A and PC‐D displaying a more pronounced contribution, reaching 90% in one case. SBS18 was present in one patient and is discussed in more detail below.

A total of 821 CNVs were identified in the four patients (Fig. 1C), with copy number losses (median, 91; range, 20–121) predominating over copy number gains (median, 62.5; range, 5–200), and copy‐neutral LOH (median, 26.5; range 20–95) was observed in all four samples, along with genome‐wide LOH in PC‐D. Recurrent copy number losses affected chromosomes 1, 3, 6, 9 and 13, and recurrent gains affected chromosomes 1, 5, 7 and 20. A common loss encompassing chromosome 3q was detected in three samples. Features of genomic instability were detected in three samples, with whole‐genome doubling in two patients and HRD, defined by LOH‐HRD score and LSTs, in one patient. All cases were microsatellite‐stable as determined using the MSIsensor, with results below the cut‐off of 3.5 (Table S3).

**Fig. 2:**
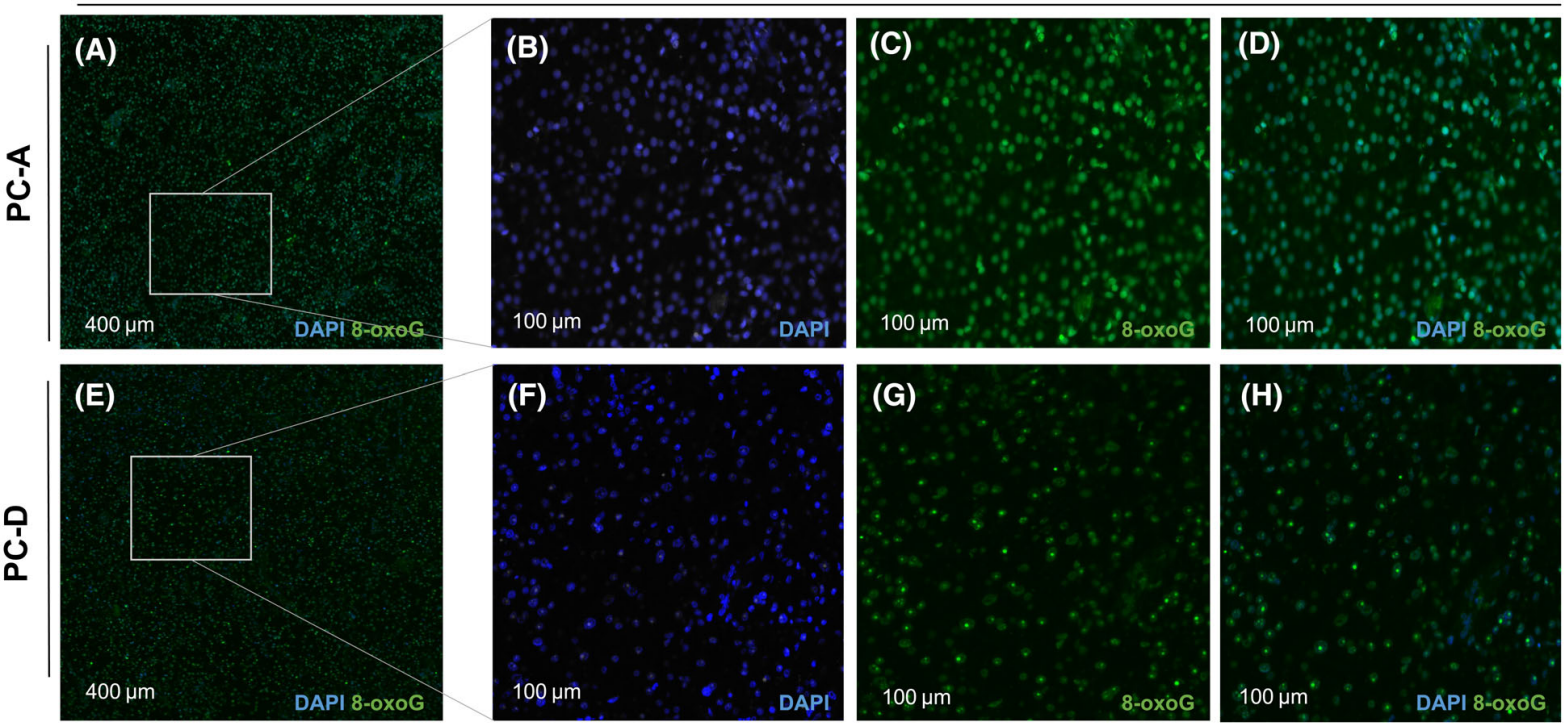
Immunofluorescence microscopic detection of 8‐oxoG in archived tissue specimens. (A, E) Overlay images of DAPI (blue) and 8‐oxoG (green) staining at low magnification (scale bar 400 μm). At higher magnification (scale bar 100 μm), detection of intense nucleolar staining of 8‐oxoG (B, F: DAPI; C, G: 8‐oxoG; D, H: overlay) in the heterozygous *MUTYH* mutation carrier PC‐D (F–H) but not in a *MUTYH*‐wildtype specimen from PC‐A (B–D). DAPI, 4′,6‐diamidino‐2‐phenylindole; 8‐oxoG, 8‐oxo‐guanine.

The four patients harboured 13 rare germline alterations affecting eight known cancer predisposition genes [32] (Table S3). One of them, a heterozygous *MUTHY* p.G393D variant in PC‐D, was classified as pathogenic. *MUTYH* encodes the DNA glycosylase mutY, which removes adenine residues mispaired with 8‐oxo‐deoxyguanosine or ‐guanine. PC‐D also had somatic LOH at *MUTYH*, resulting in biallelic inactivation of *MUTYH*, a constellation conferring a heritable predisposition to colorectal carcinoma and possibly other malignancies termed *MUTYH*‐associated polyposis. Interestingly, this sample also displayed SBS18, most likely caused by the *MUTYH* variant and somatic LOH [33, 34]. Immunohistochemistry showed increased 8‐oxo‐guanine staining with a predominant nucleolar pattern in archived tumour tissue but not in the control sample (Fig. 2).

### 3.2 Kinase expression landscape

**Fig. 3:**
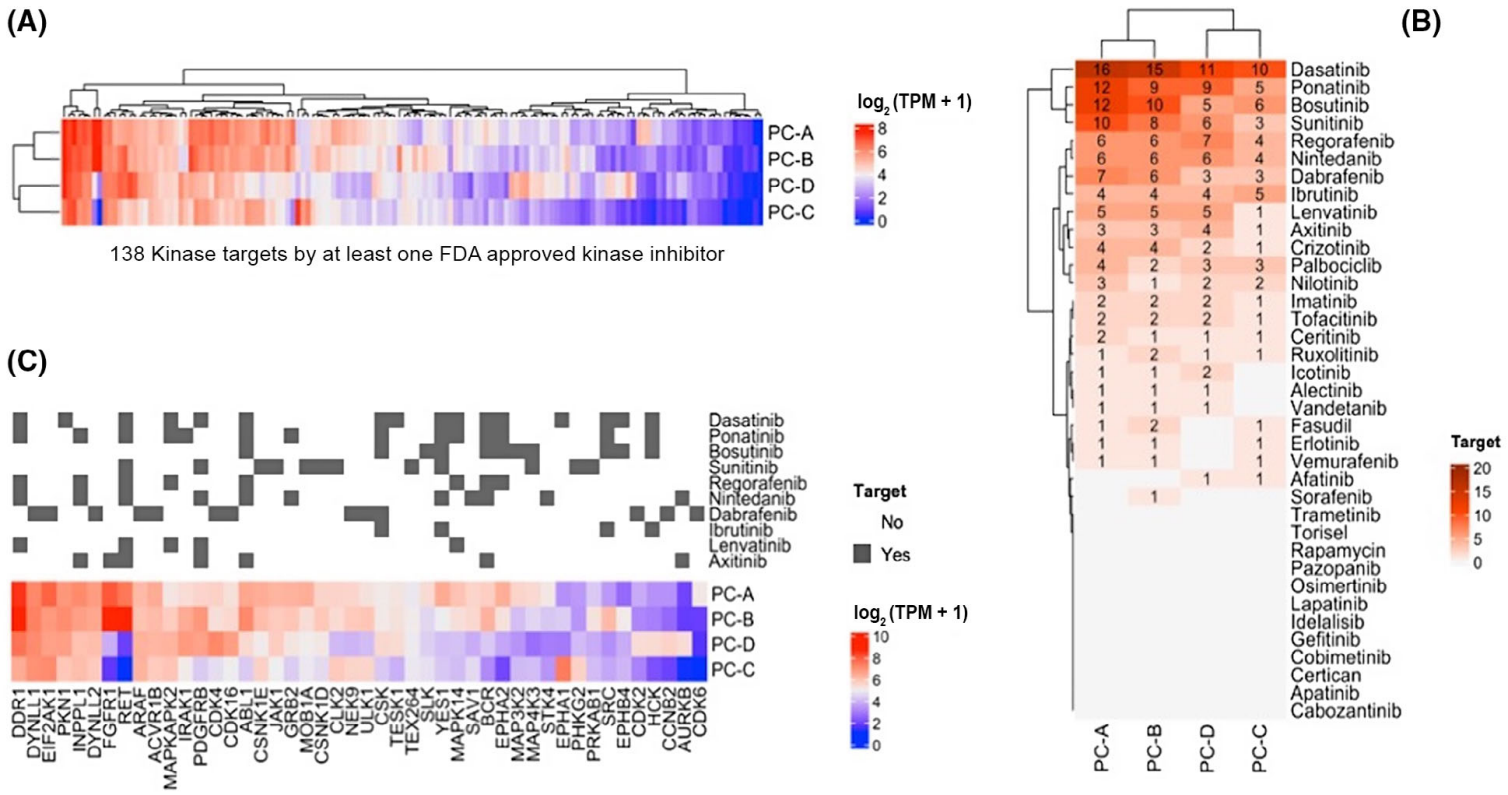
Kinase expression landscape of PC. (A) mRNA expression of 138 kinases targeted by at least one of 37 FDA‐approved small‐molecule inhibitors in the four PC samples. (B) Ranking of kinase inhibitors by the number of targets expressed in the top 25%. (C) Drug–target matches for kinase inhibitors with at least five overexpressed targets in at least two samples. Note that for PC‐B, which showed both *FGFR1* and *RET* overexpression, only *RET* was predicted as a lenvatinib target. FDA, United States Food and Drug Administration.

To identify aberrant activation of potentially druggable signalling pathways, we examined the expression patterns of 138 kinases that can be targeted by at least one small‐molecule inhibitor approved by the United States Food and Drug Administration (FDA). We noticed striking overexpression of *RET* and *FGFR1* in PC‐A and PC‐B compared with PC‐C and PC‐D (Fig. 3A,C).

Considering the top 25% expressed kinase genes across the four samples, seven kinase inhibitors with at least five targets in at least one sample were identified as potential therapies (Fig. 3B,C). Dasatinib, ponatinib and bosutinib are biomarker‐based treatments for chronic myeloid leukaemia and Philadelphia chromosome‐positive acute lymphoblastic leukaemia [35, 36, 37, 38]. Dabrafenib is a mutant *BRAF*‐specific inhibitor approved for melanoma and non‐small‐cell lung cancer. Nintedanib has activity against VEGFR, FGFR and PDGFR and is approved for non‐small cell lung cancer in combination with docetaxel [39]. Ibrutinib is a BTK inhibitor approved for the treatment of lymphatic neoplasms [40]. Lenvatinib primarily targets VEGFR and FGFR family members and, to a lesser extent, RET and KIT and is approved as monotherapy for the treatment of hepatocellular carcinoma and differentiated thyroid cancer [41, 42].

To explore changes in pathway activity that cannot be inferred from the expression of individual kinase genes, we further used the PROGENy algorithm. An enrichment of PIK3CA, MAPK and WNT signalling was observed in PC‐A, whereas PC‐D showed upregulation of the NFkB, TNFa, JAK‐STAT, VEGF and Trail pathways (Fig. S3).

### 3.3 Genomics and transcriptomics‐guided treatment

In the MASTER program, biomarkers and treatment recommendations are categorized into eight molecular intervention baskets based on the cellular pathways or processes involved: tyrosine kinases (TKs), PI3K‐AKT‐mTOR (PAM), RAF‐MEK‐ERK (RME), cell cycle (CC), developmental regulation (DEV), DNA damage repair (DDR), immune evasion (IE) and other (OTH).

At least two targeted therapies were recommended for each patient. These were assigned to the IE (three patients), TK (three patients), PAM (two patients), DDR (two patients), DEV (one patient) and CC (one patient) baskets. Treatment combinations were recommended in two cases (Table S3). In two patients, molecularly guided treatment was implemented.

PC‐A, a 43‐year‐old woman with locally advanced PC, remained in remission for 12 years after primary treatment when she developed abdominal LN metastases. Based on a high TMB (496 functional somatic SNVs and seven functional somatic indels) and the APOBEC mutational signatures SBS2 and SBS13 in a metastasis sample, the PD‐1 inhibitor pembrolizumab was recommended. Interestingly, compared with the other three cases, the CIBERSORT deconvolution algorithm, an immune infiltration analysis tool in immunedeconv, indicated high fractions of activated CD8‐positive T cells, T‐follicular helper cells, inflammatory M1 macrophages and a small fraction of regulatory T cells (Tregs), suggesting a responder phenotype for immune checkpoint inhibitor treatment [43] (Fig. S4). Pembrolizumab, initiated on a compassionate‐use basis, resulted in disease stabilization for 12 months and a clear biochemical response [44].

**Fig. 4:**
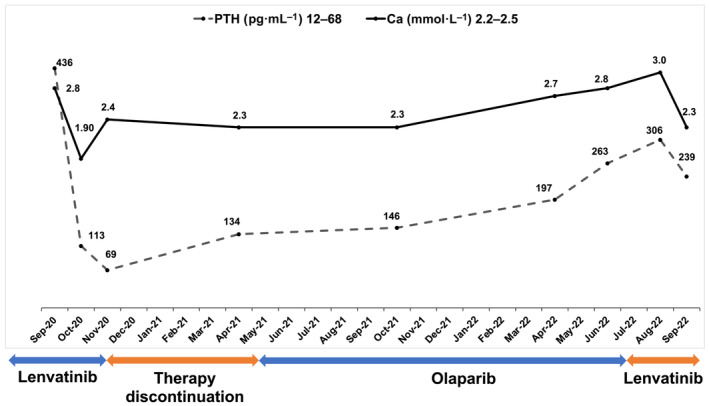
Biochemical response of patient PC‐B to lenvatinib and olaparib. Tumour progression with parathyroid hormone increased to 436 pg·mL^−1^ (reference range, 12–68 pg·mL^−1^) and moderate hypercalcemia (2.8 mmol·L^−1^; reference range, 2.2–2.5 mmol·L^−1^) despite cinacalcet treatment was observed. Administration of the TK inhibitor lenvatinib at a dose of 24 mg decreased parathyroid hormone to 113 pg·mL^−1^ within 3 weeks, and symptomatic hypocalcemia of 1.9 mmol·L^−1^ with paresthesias and muscle cramps occurred, requiring replacement of calcium citrate and activated vitamin D. Lenvatinib was discontinued because of renal impairment. Five months later, radiologic and biochemical progression occurred. Further molecularly informed treatment with the PARP inhibitor olaparib was started, resulting in disease stabilization as the best response. After 14 months, olaparib was discontinued due to disease progression, and lenvatinib was reintroduced, resulting in another biochemical response. PTH, parathyroid hormone; Ca, calcium.

Patient PC‐B, a 51‐year‐old man with localized PC, relapsed 4 years after primary resection with lung and skull base metastases, for which he underwent repeat surgical interventions. Rapid progression required radiation to the skull base. Molecular analysis of a skull base metastasis revealed overexpression of *FGFR1* and *RET*, which prompted treatment with lenvatinib. The patient experienced complete biochemical remission and remained on therapy for 3 months. Due to adverse events, treatment was discontinued, and radiologic and biochemical disease progression occurred 5 months later. The second molecularly informed treatment with the PARP inhibitor olaparib, informed by somatic loss of *BRCA2* and detection of SBS3 [45], resulted in disease stabilization lasting 14 months (Fig. 4) before lenvatinib was resumed.

Patient PC‐C was a 39‐year‐old man who underwent left parathyroidectomy and hemithyroidectomy followed by remission for 13 years. He subsequently developed local relapse with LN metastases, for which he underwent multiple surgical procedures before enrolling in the MASTER program. He was lost to follow up, and no information is available on treatment or response.

Finally, patient PC‐D was an 82‐year‐old woman with localized PC who developed lung metastases 1 year after the initial surgery. High expression of somatostatin receptor 5 detected by immunohistochemistry prompted two courses of peptide receptor‐mediated radionuclide therapy with Lu‐177 DOTATOC, but this did not halt the progression of LN metastases. A surgical biopsy of one LN was subjected to molecular analysis, but the patient died of progressive disease before targeted therapy could be considered. Also in this patient, immune checkpoint inhibition was recommended based on high TMB, although immune cell deconvolution suggested a protumoral tumour microenvironment characterized by higher proportions of Tregs and immunosuppressive M2 macrophages and fewer M1 macrophages, CD8‐positive T cells and T‐follicular helper cells (Fig. S4).

## 4 Discussion

This is the first study to perform genome‐ and transcriptome‐wide characterization of advanced PC to guide clinical management of patients with this ultra‐rare cancer after failure of standard therapy. Analyses were performed using the standardized workflow established in the NCT/DKFZ/DKTK MASTER program for young adults with advanced cancers and patients with refractory rare malignancies [15]. Molecularly informed treatment recommendations could be made in all four cases, and biochemical response and prolonged disease stabilization were observed in both patients in whom treatment was implemented.

Both DNA and RNA sequencing provided clinically valuable information. Because cryopreserved tumour tissue is required for whole‐genome and RNA sequencing, as used in our study, a direct comparison with archival tissue samples from primary and metastatic lesions to shed light on spatial and temporal heterogeneity in PC was not possible. However, previous targeted RNA expression studies on formalin‐fixed, paraffin‐embedded samples have revealed marked differences in gene expression between primary tumours and metastases from different patients [46].

In line with previous data, we detected somatic *CDC73* mutations in two of four patients, which have been linked to a more aggressive disease course and increased TMB [14], features that we also observed in our patients. We also confirmed amplification and concomitant overexpression of *CCND1* as a recurrent alteration in PC [8], suggesting that a subset of PC depends on the deregulation of this druggable pathway and, in one case, triggering the recommendation of a CDK4/6 inhibitor. Deleterious *TP53* mutations are rare in PC and associated with a more aggressive clinical course [8]. Consistent with this, the patient with *TP53*‐mutant PC in our cohort relapsed after 1 year with rapid clinical deterioration. The pathogenetic role and potential clinical significance of additional tumour suppressor genes in PC are largely unknown. Our observation of deletion of chromosome 3q in three of four samples suggests that one or more such genes may be located in this region.

The increased TMB observed in two cases could be attributed to two different genetic mechanisms. In PC‐D, LOH for a heterozygous germline mutation in *MUTYH* was observed in the tumour and associated with the characteristic SBS18, supported by evidence of increased oxidative DNA damage on immunohistochemistry. While biallelic germline mutations in *MUTYH* are associated with adenomatous polyposis and increased risk of colorectal cancer, the relative cancer risk for heterozygous carriers is less clear. Monoallelic *MUTYH* variants contribute to tumorigenesis through loss of the remaining functional *MUTYH* allele via LOH. The characteristic C‐to‐A base substitutions in *MUTYH*‐deficient tumours increase the formation of stop codons, contributing to the inactivation of tumour suppressor genes [47, 48]. The copy‐neutral LOH and whole‐genome doubling in our patient resemble the pattern observed in adrenocortical carcinoma, which has been reported as the most frequent cancer in heterozygous *MUTYH* carriers [32, 49] and points to a possible role for *MUTYH* in the pathogenesis of this PC case.

In addition, PC‐D, the second patient with elevated TMB, exhibited SBS2 and SBS13, which are attributed to APOBEC cytidine deaminase activity and account for more than one‐third of human cancers signatures [14, 22]. As SBS2 and SBS13 have been associated with response to immune checkpoint inhibition [50], they were among the biomarkers considered for treatment recommendation in both cases. PC‐A benefited from the PD‐1 antagonist pembrolizumab with a biochemical response and prolonged disease stabilization and has been tumour‐free and without biochemical evidence of disease for more than 2 years after repeat surgery.

Thus, the clinical courses of PC‐A and PC‐D underscore the clinical utility of integrating composite biomarkers – in these cases, TMB and mutational signatures – in the workup of advanced PC. This general strategy, which requires comprehensive molecular profiling, is also supported by patient PC‐C, in whom evidence of HRD and HRD‐LOH provided a rationale for PARP inhibition, which was implemented after treatment with lenvatinib and led to prolonged disease stabilization.

Recent studies have demonstrated that transcriptome analysis can substantially increase the number of patients treated in molecularly informed clinical trials [51, 52]. Accordingly, RNA‐seq has been shown to significantly expand molecularly informed treatment options in the MASTER trial with particular utility in the TK basket [15]. In the current analysis, we interrogated the RNA‐seq results beyond detecting oncogenic fusion genes and aberrantly expressed single genes and used experimental data on inhibitor activity towards various TKs to derive expression patterns predictive of response. By matching the kinase genes overexpressed in the four tumours with the target spectrum of FDA‐approved inhibitors, we identified dasatinib and ponatinib as the compounds with the most targets in our patients, that is, with inhibitory activity against five to 16 overexpressed kinase genes. Furthermore, in PC‐A and PC‐B, overexpression of *FGFR1* and *RET* prompted the recommendation of treatment with lenvatinib by the MTB. This led to a dramatic biochemical response in PC‐B, even entailing hypocalcemia as an indicator of response, and morphologic disease stabilization for 8 months. In this exceptional responder, only RET but not FGFR1 was among the targets of lenvatinib when only the top 25% expressed kinases with a high binding affinity (threshold, 1 μm) were considered. Together with the strong overexpression of *RET* in PC‐A, these observations suggest a role for RET signalling in the development and/or progression of a subset of PC, a hypothesis that requires further validation in preclinical models and the clinical setting. Notably, a previous study comparing parathyroid adenoma, non‐metastatic and metastatic PC found that *FGFR1* is one of the genes overexpressed in metastatic versus non‐metastatic PC, also emphasizing FGFR1 inhibition as a potential therapeutic strategy [46].

## 5 Conclusions

In summary, our findings reveal novel aspects of the molecular pathogenesis of PC, particularly the importance of mutational processes reflected in the APOBEC signatures SBS2 and SBS13, heterozygous germline mutations in *MUTYH* as potential predisposing factors, and recurrent overexpression of *FGFR1* and *RET*. Notably, some of these features provide novel therapeutic targets. Our work thus underscores the scientific and clinical value of multi‐omics‐based and entity‐spanning precision oncology programs, which is particularly apparent in very rare and poorly understood cancers for which there are few standards of care, such as PC.

## Conflict of interest

C. Terkamp: Consulting or advisory board membership: AstraZeneca, Lilly, NovoNordisk; honoraria: AstraZeneca, Lilly, Merck Sharp and Dohme, NovoNordisk, Takeda; travel or accommodation expenses: AstraZeneca, Ipsen, Lilly, NovoNordisk, Pfizer. S. Fröhling: Consulting or advisory board membership: Bayer, Illumina, Roche; honoraria: Amgen, Eli Lilly, PharmaMar, Roche; research funding: AstraZeneca, Pfizer, PharmaMar, Roche; travel or accommodation expenses: Amgen, Eli Lilly, Illumina, PharmaMar, Roche. M. Kroiss: Consulting or advisory board membership: Advanz, Bayer, Eisai, Eli Lilly; research funding: Eli Lilly, Ipsen, Loxo; honoraria: Eli Lilly, Ipsen, Merck Sharp and Dohme, Roche, Sanofi; travel or accommodation expenses: Eli Lilly. The other authors declare no competing interests.

## Author contributions

SF and MK designed the study. M‐VT, CTF, NP, SP, AM, SK, L‐SL, AJ, and DH performed experiments and analysed data. AM, CT, CL, SK, NS, AS, MF, HG, SF, and MK contributed to collection and interpretation of data. M‐VT, CTF, SF, and MK drafted the manuscript. All authors critically revised and contributed to the manuscript and approved its final version.

## Supporting information


**Fig. S1.** Genomic landscape of PC.
**Fig. S2.** Expression of *CCND1*, *RET*, *FGFR1* in samples PC‐A, B, C, and D compared to 149 background samples from the NCT/DKFZ/DKTK MASTER cohort.
**Fig. S3.** RNA‐based pathway activity inference using the PROGENy algorithm.
**Fig. S4.** Immune cell infiltration.
**Table S1.** Kinase gene expression.
**Table S2.** Kinase gene outlier expression.
**Table S3.** Raw and processed patient‐individual sequencing results.

## Data Availability

The data supporting the findings of this study are available in Tables S1–S3. Genomic and transcriptomic data have been deposited in the European Genome‐Phenome Archive (https://www.ebi.ac.uk/ega/datasets) under accession EGAD00001000002.
