# Supplementary material for: Targeted therapy of advanced parathyroid carcinoma guided by genomic and transcriptomic profiling
Source: Mol Oncol. 2023 Apr 11;17(7):1343–55. doi: 10.1002/1878-0261.13398 (PMC10323885; doi:10.1002/1878-0261.13398)

Fig. S1. Genomic landscape of parathyroid carcinoma

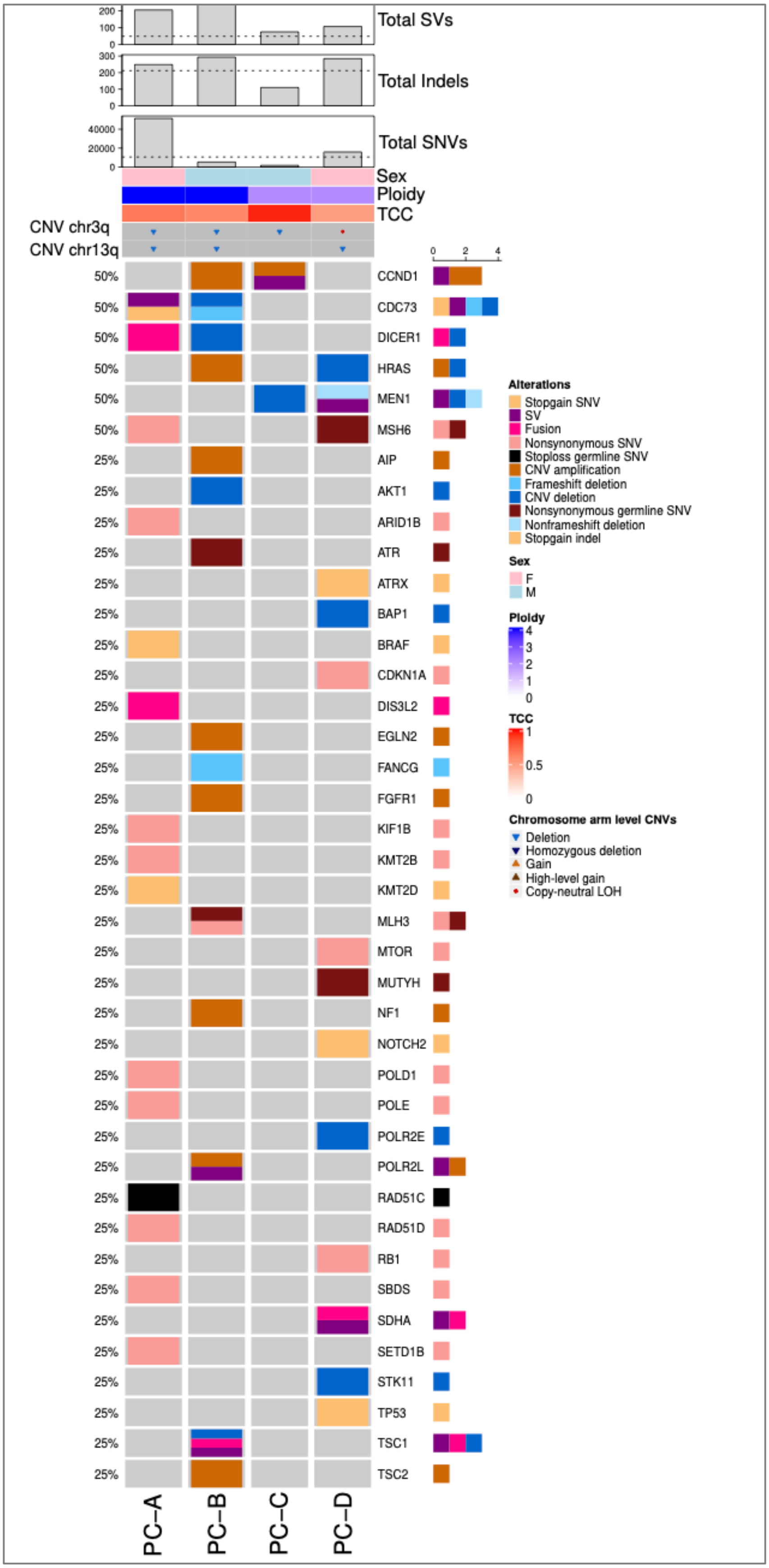

Fig. S2. Expression of *CCND1*, *RET*, *FGFR1* in samples PC-A, B, C, and D compared to 149 background samples from the NCT/DKFZ/DKTK MASTER cohort.

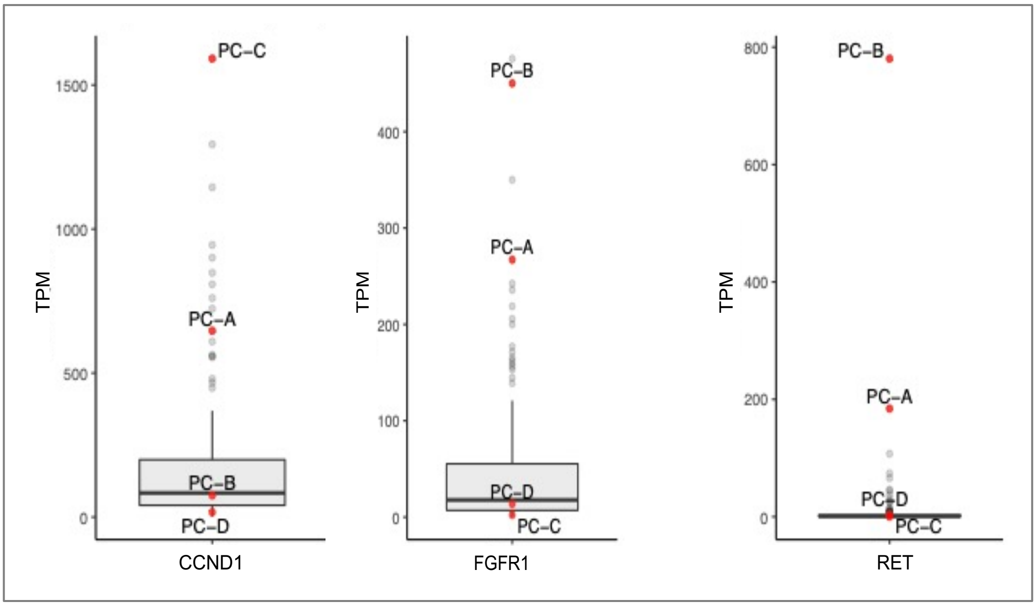

Fig.S3. RNA based pathway activity inference using the PROGENy algorithm.

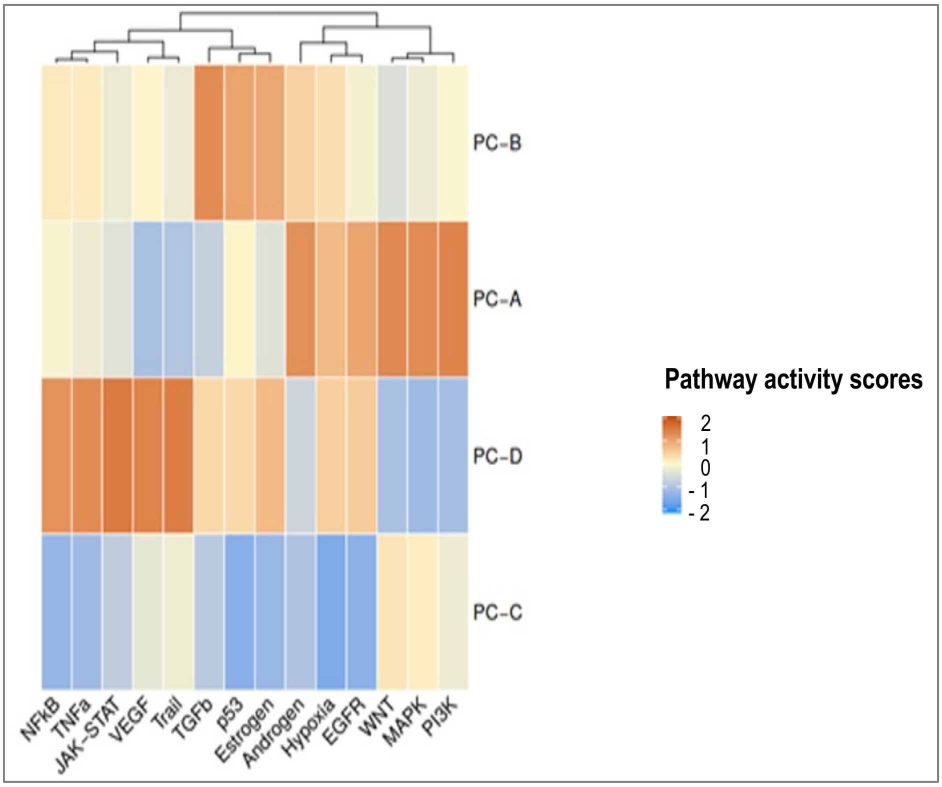

Fig. S4. Immune cell infiltration

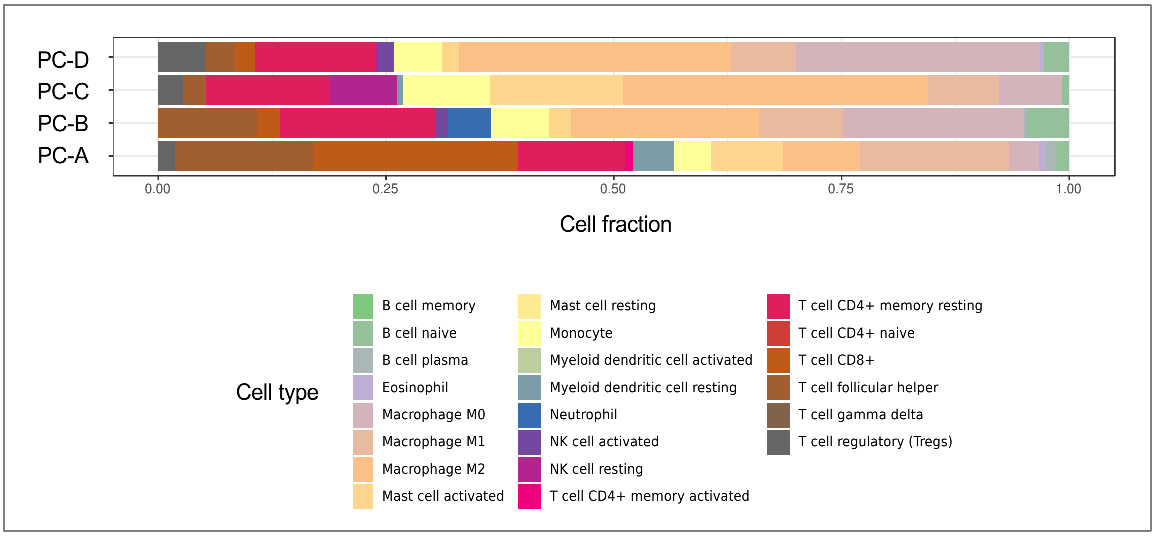

Supplement: Supplementary file 1 — Fig. S1. Genomic landscape of PC. Fig. S2. Expression of CCND1, RET, FGFR1 in samples PC‐A, B, C, and D compared to 149 background samples from the NCT/DKFZ/DKTK MASTER cohort. Fig. S3. RNA‐based pathway activity inference using the PROGENy algorithm. Fig. S4. Immune cell infiltration. Table S1. Kinase gene expression. Table S2. Kinase gene outlier expression. Table S3. Raw and processed patient‐individual sequencing results. [file MOL2-17-1343-s001.zip › mol213398-sup-0001-FigsS1-S4.pdf]
