# Supplementary material for: Targeted therapy of advanced parathyroid carcinoma guided by genomic and transcriptomic profiling
Source: Mol Oncol. 2023 Apr 11;17(7):1343–55. doi: 10.1002/1878-0261.13398 (PMC10323885; doi:10.1002/1878-0261.13398)
Supplement: Supplementary file 1 — Fig. S1. Genomic landscape of PC. Fig. S2. Expression of CCND1, RET, FGFR1 in samples PC‐A, B, C, and D compared to 149 background samples from the NCT/DKFZ/DKTK MASTER cohort. Fig. S3. RNA‐based pathway activity inference using the PROGENy algorithm. Fig. S4. Immune cell infiltration. Table S1. Kinase gene expression. Table S2. Kinase gene outlier expression. Table S3. Raw and processed patient‐individual sequencing results. [file MOL2-17-1343-s001.zip › mol213398-sup-0004-Legends.docx]

**Targeted therapy of advanced parathyroid carcinoma guided by genomic and transcriptomic profiling**

**Supporting Information**

**Fig. S1.** **Genomic landscape of PC.** Mutations occurring in at least one of the known PC driver genes and a set of 143 germline predisposition genes.

**Fig. S2. Expression of *CCND1*, *RET*, *FGFR1* in samples PC-A, B, C, and D compared to 149 background samples from the NCT/DKFZ/DKTK MASTER cohort.**

**Fig. S3.** **RNA-based pathway activity inference using the PROGENy algorithm.** Enhanced activity of the PI3K, MAPK, and WNT signaling pathways in the sample from patient PC-A and of the NFkB, TNFa, JAK-STAT, VEGF, and Trail pathways in the sample from patient PC-D.

**Fig. S4. Immune cell infiltration.** The sample from patient PC-A showed a high proportion of activated CD8-positive T cells, T-follicular helper cells, and inflammatory M1 macrophages and a low proportion of regulatory T cells (Tregs), consistent with an inflammatory immunophenotype with antitumor activity in the microenvironment. In contrast, PC-D was characterized by an immunologically quiet immunophenotype exhibiting higher proportions of Tregs and immunosuppressive M2 macrophages, fewer M1 macrophages, and lower proportions of CD8-positive T cells and T-follicular helper cells. In PC-B and PC-C, the proportion of activated lymphocytes was low, but both samples had higher proportions of M2 macrophages, which have been associated with tumor progression and metastasis.

**Supplementary Table 1. Kinase Gene Expression**

**Supplementary Table 2. Kinase Gene Outlier Expression**

SF2.1. Tyrosine Kinases

SF2.2. Druggable Kinases

SF2.3. All Kinases

**Supplementary Table 3**

SF3.1. SNVs

SF3.2. Indels

SF3.3. Germline SNVs and Indels

SF3.4. PC Driver Genes

SF3.5. Germline Predisposition Genes

SF3.6. Mutational Signatures

SF3.7. HRD Scores and MSIsensor Results

SF3.8. Treatment Basket Recommendations
